# Decreased outlet angle of the superior cerebellar artery as indicator for dolichoectasia in late onset Pompe disease

**DOI:** 10.1186/s13023-018-0794-6

**Published:** 2018-04-13

**Authors:** Ole Hensel, Ilka Schneider, Mathias Wieprecht, Torsten Kraya, Stephan Zierz

**Affiliations:** 1 Department of Neurology, University hospital Halle/Saale, Ernst-Grube-Str. 40, 06120 Halle/Saale, Germany; 2 Department of Diagnostic Radiology, University hospital Halle, Ernst-Grube-Str. 40, Halle/Saale, Germany

**Keywords:** Late onset Pompe disease (LOPD), Dolichoectasia of basilar artery, Dilative arteriopathy, Height of basilar bifurcation, Glycogenosis type II, Superior cerebellar artery (SUCA, SCA), Acid maltase deficiency

## Abstract

**Background:**

Lysosomal α-glucosidase deficiency (Pompe disease) not only leads to glycogen accumulation in skeletal muscle, but also in the cerebral arteries. Dolichoectasia of the basilar artery (BA) has been frequently reported. Therefore progression of BA dolichoectasia in late onset Pompe patients (LOPD) was studied.

**Methods:**

BA length, diameter and volume, and cerebral lesions were analysed by MRI/TOF-MR angiography or CT/CT angiography in 20 LOPD patients and 40 controls matching in age, sex- and cardiovascular risk factors. The height of BA bifurcation was assessed semi-quantitatively using the Smoker’s criteria and quantitatively by measuring the outlet angle of the superior cerebellar artery (SUCA). Nine patients were followed over 5 years.

**Results:**

The height of the BA bifurcation was abnormal in 12/20 (60%) LOPD patients and in 12/40 (30%) matched controls. The SUCA outlet angle was reduced in LOPD patients compared to controls (127 ± 33° vs. 156 ± 32°, *p* = 0.0024). The diameter, length and volume of the BA were significantly increased in LOPD patients compared to controls. 12/20 (60%) LOPD patients and 27/40 (68%) controls presented white matter lesions. During 5 years 2/9 LOPD patients developed an abnormal height of BA bifurcation according to the Smoker’s criteria and in all patients the SUCA outlet angle decreased (138 ± 34° vs. 128 ± 32°, *p* = 0.019). One patient with prominent basilar dolichoectasia experienced a thalamic hemorrhage.

**Conclusion:**

Pompe disease is associated with BA dilation, elongation and elevated bifurcation height of the BA which might result in cerebrovascular complications. The SUCA outlet angle seems to be useful for monitoring the progression of BA dolichoectasia.

**Electronic supplementary material:**

The online version of this article (10.1186/s13023-018-0794-6) contains supplementary material, which is available to authorized users.

## Background

Pompe disease (glycogenosis type II, OMIM# 232300) is a multi-systemic disorder caused by reduced activity of the lysosomal acid glycosidase α presenting with predominant muscular weakness, respiratory and cardiac insufficiency [1]. Autopsy studies showed glycogen accumulation also in smooth muscles of the tunica media of cerebral arteries indicating involvement of the cerebrovascular system [2]. Microangiopathies (e.g. white matter lesions, microbleeds) and macroangiopathies (e.g. dolichoectasia and aneurysms mostly of vertebrobasilar arteries) have been repeatedly reported in late onset Pompe disease (LOPD) [3]. Cerebrovascular complications might be crucial for long term survival of LOPD patients as these cerebrovascular changes were associated with intracranial haemorrhages, compression of cranial nerves and brainstem, and stroke [3–6]. Recent studies have demonstrated that dolichoectasia of the basilar artery (BA) is the most striking cerebrovascular finding in LOPD patients [3]. A preliminary study showed a significant dilation of vertebrobasilar arteries compared to controls [7]. However, little is known about the progression of this arterial remodelling in the course of LOPD. Therefore this study investigated how BA characteristics of 20 LOPD patients differ from those of 40 subjects that individually matched the LOPD patients in age-, sex-, and risk factors. Secondly the progression of BA arteriopathy during a 5 year observation period was analysed retrospectively for 9 of the 20 LOPD patients. Finally the occurrence of cerebral lesions was examined.

### Patients and controls

Twenty LOPD patients with genetically and biochemically confirmed diagnosis of Pompe disease (age 53.7 ± 14.6 years, range 19 to 81) were studied. All Patients were on enzyme replacement therapy (ERT) (mean 68.8 ± 42.8 months; range 9 to 135 months). Table 1 summarizes clinical and molecular data of LOPD patients. Follow up MRI examination was performed after 5 years in 9/20 LOPD patients.

**Table 1:** Clinical and molecular data of 20 LOPD patients

Pat.-Nr.	Sex	*GAA*-Genotype		Age [years]	Duration disease (years)	Duration ERT (month)	Cardio-vascular risk factors
P1^a^	F	IVS1 (− 13 T > G)	IVS9 G > C)	26	15	67	HL
P2	F	p.L552P	p.P493L	37	10	33	HL, O
P3^a^	M	IVS1 (−13 T > G)	c.2136-7delGT	50	40	116	HYP, HL
P4^a^	M	IVS1 (−13 T > G)	p.W499R	50	18	130	DM, HL
P5^a^	M	IVS1 (−13 T > G)	p.P493L	52	15	63	HYP, HL
P6^a^	F	IVS1 (−13 T > G)	p.L552P	53	17	99	HYP, HL, O, S
P7^a^	M	IVS1 (−13 T > G)	p.C103G	59	14	135	HYP, DM, HL, O
P8^a^	F	IVS1 (−13 T > G)	p.P493L	61	21	63	HYP, HL
P9	M	IVS1 (−13 T > G)	p.G309R	64	11	14	HYP, DM, O
P10^a^	F	IVS1 (−13 T > G)	del exon 18	75	40	103	HYP
P11	M	IVS1 (−13 T > G)	c.832delC	45	6	17	HYP, HL
P12	F	IVS1 (−13 T > G)	c.525delT	66	7	33	HYP, HL
P13	M	IVS1 (−13 T > G)	IVS1 (−13 T > G)	61	13	29	HYP, HL
P14	F	IVS1 (−13 T > G)	c.2481 + 102_2646 + 31del	54	18	44	HYP, HL
P15	F	IVS1 (−13 T > G)	c.2481 + 102_2646 + 31del	56	19	48	HYP, HL
P16^a^	F	IVS1 (−13 T > G)	c307 T > G	49	23	119	HL
P17	M	IVS1 (−13 T > G)	p.G309R	81	6	37	HYP, HL
P18	M	IVS1 (−13 T > G)	c.794delG	49	13	84	NONE
P19	F	IVS1 (−13 T > G)	c.925G > A	61	2	9	HYP
P20	M	IVS1 (−13 T > G)	c.525delT	19	19	132	NONE

Patient P1-P10 were numbered according to their number in the previous study by Hensel et al. [7]. P5 + P8 and P14 + 15 were siblings; a patients with 5 years follow-up; Abbr.: *ERT* enzyme replacement therapy, *GAA* Glucosidase alpha gene. Risk factors: *DM* diabetes mellitus, *HL* hyperlipidemia, *HYP* Hypertension, *O* obesity, *S* smoking

For each Pompe patient two controls (*n* = 40; age 54.0 ± 14.7 years, range 19 to 80) were recruited that matched in gender, age (maximal ±3 years) and number of cerebrovascular risk factors (maximal ±1). Relevant risk factors were defined as hypertension, hyperlipidemia, obesity, smoking and diabetes. The control group consisted of patients with other neurological diseases that underwent cerebral MRI imaging for diagnostic purpose. This included headache (11/40), TIA and minor cardioembolic stroke (7/10), peripheral neuropathies (5/40), seizures (4/40), myopathies other than Pompe disease (3/40), other diagnoses (10/40) were retrobulbar optic neuritis, anterior ischemic optic neuropathy, spinocerebellar syndrome, dementia, and psychiatric diseases.

## Methods

**Fig. 1:**
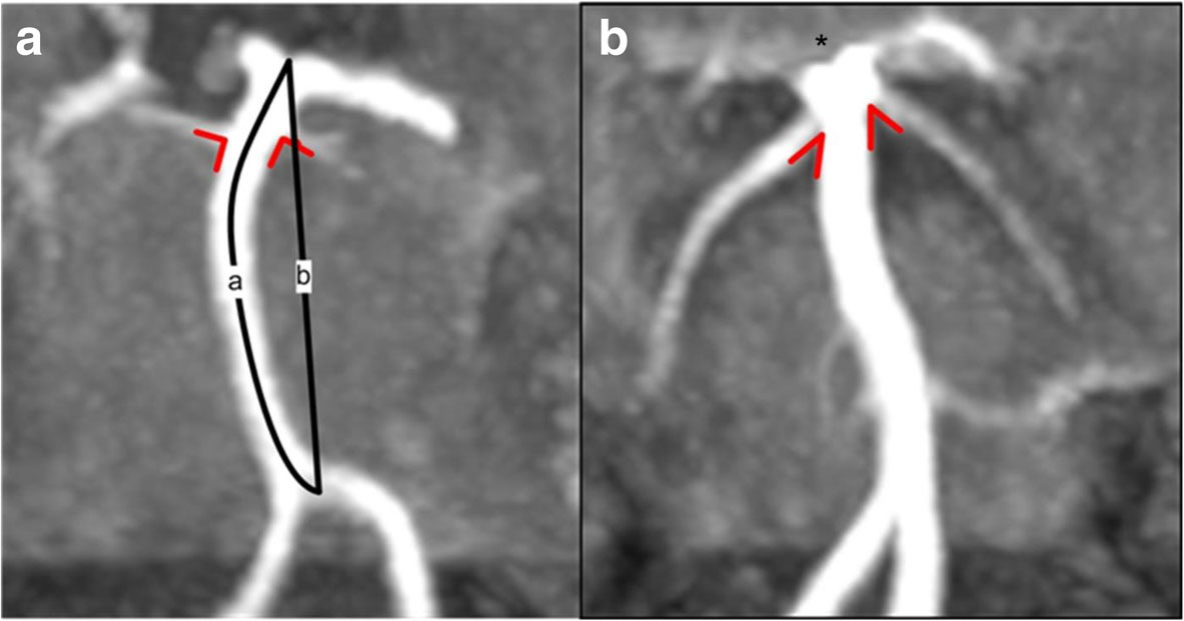
Basilar artery (BA) and the outlet angle of the superior cerebellar artery (SUCA) Assessment of the BA length and bifurcation height and SUCA outlet angle. **a**) A 57 year old male control patient with normal height of the BA bifurcation (grade 0 according to Smoker criteria [8]) and nearly perpendicular SUCA outlet from the basilar artery (angle right: 85°, angle left: 75°; SUCA outlet angle of 160° as defined by sum of left and right side). **b**) A 61 year old female LOPD patient with dilated basilar artery, cranial shifted BA bifurcation indenting the third ventricle and hypothalamus (*, grade 3 according to Smoker’s criteria [8]) and low SUCA outlet angle (angle right: 48°, angle left: 31°; SUCA outlet angle of 79° as defined by sum of left and right side). Schematic drawings indicate measurements of SUCA outlet angles (red angles) and measurements of basilar artery: anatomic length (line a) and linear length (line b)

All participants except one LOPD patient underwent cerebral TOF-MR angiography and FLAIR-MRI. Additional T2*-weighted sequences were performed in 19 LOPD patients (1.5 Tesla Magnetom Sonata Vision, Siemens, Erlangen, Germany). Because of cardiac pacemaker one LOPD patient was examined by cerebral CT and CT angiography. Follow up imaging was performed with the identical MRI scanner and MRI sequences. There were slight differences in the TOF-MRI protocol. All images were anonymized and reviewed by the neuroradiologist (M.W.) and by a skilled neurologist (O.H.). Smoker’s criteria for basilar dolichoectasia [8] were applied for characterisation of the BA: The semi-quantitative grading of the height of the BA bifurcation was assessed (grade 0: at or below dorsum sellae; grade 1: within suprasellar cistern; grade 2: at level of third ventricle; grade 3: indenting and elevating floor of third ventricle; abnormal ≥ grade 2) and the lateral displacement of the BA (grade 0: midline throughout; grade 1: medial to lateral margin of clivus or dorsum sellae; grade 2: lateral to lateral margin of clivus or dorsum sellae; grade 3: in cerebellopontine angle cistern; abnormal ≥ grade 2). The inner diameter of BA was quantified proximally (1 mm after BA origin), at the middle (half of the anatomic BA length), and distally (3 mm before BA bifurcation). The smallest diameter on axial TOF-MR images was used. For calculation of BA volume the smallest diameter was measured in 1 mm slices. For all diameter measurements, an intensity–based analysis was used (FWHM Line-plugin of ImageJ-Software, version 1.51 h, NIH, USA). This allowed a precise and user independent diameter measurement [9]. The anatomical and the linear length of the BA were measured from the conjunction of both vertebral arteries (VA) to the BA bifurcation (as depicted in Figure 1a). Additionally the outlet angle of the superior cerebellar artery (SUCA) from the BA was assessed using hypotenuse (SUCA) and adjacent side (BA) at 5 mm distance (as depicted in Figure 1). The SUCA outlet angle was defined as the sum of the left and the right outlet angle. This method was chosen to take into account that the SUCA outlet might differ between left and right side because of a frequent asymmetry of the SUCA. In FLAIR-MRI, the deep white matter lesions were graded according to the Fazekas score [10]. The gradient-echo T2-weighted (T2*) images were screened for cerebral microbleeds. Statistical analysis was conducted using SigmaStat (SYSTAT Software GmbH, version 4.0, Germany). A *p*-value < 0.05 was considered significant. The research protocol was approved by the Ethical Committee of the medical faculty of the Martin-Luther-University Halle-Wittenberg.

## Results

### BA characteristics in 20 LOPD patients and 40 controls

The height of the BA bifurcation was abnormal in 12/20 LOPD patients (60%) and in 12/40 matched controls (30%). Abnormal lateral displacement of the BA was observed in 1/20 LOPD patients (5%) and in 1/40 matched controls (2.5%). LOPD patients had significantly dilated middle and distal BA diameters compared to matched controls. The anatomic length, linear length, and volume of the BA in LOPD patients were also significantly higher than in the control group. The SUCA outlet angles were significantly reduced in LOPD patients compared to controls (Table 2).

**Table 2:** Characteristics of basilar artery in LOPD and controls measured by TOF-MRA

	LOPD (*n* = 20)	controls (*n* = 40)	*p*-value ‡
Basilar artery characteristics			
- Diameter (mm)			
• proximal • middle • distal	3.5 ± 0.8 3.2 ± 1.2 3.2 ± 1.1	3.1 ± 1.0 2.4 ± 0.4 2.7 ± 0.6	*p* = 0.130 *p* = 0.0006 *p* = 0.013
- Anatomical length (mm)	34.0 ± 6.6	30.7 ± 5.2	*p* = 0.039
- Linear length (mm)	31.3 ± 3.7	29.1 ± 4.0	*p* = 0.051
- Volume (ml)	0.209 ± 0.164	0.113 ± 0.547	*p* = 0.0014
Smoker criteria [8]			
- Abnormal lateral BA displacement (≥ grade 2)	1/20 (5%)	1/40 (2.5%)	*p* = 1.00
- Abnormal height of BA bifurcation (≥ grade 2)	12/20 (60%)	12/40 (30%)	*p* = 0.049
Other parameter			
SUCA outlet angle (°)	127 ± 33^a^	156 ± 32	*p* = 0.0024
White matter lesions	12/19 (63%)^b^	27/40 (68%)	*p* = 0.77

**‡** application of two sample t-test; ^a^ in one LOPD patient the BA bifurcation was such abnormal, that SUCA outlet angle was not measurable; ^b^ one LOPD patient had a CT

In LOPD patients, there was a positive correlation between BA anatomical length (Pearson *r* = 0.626, *p* = 0.0.003), linear length (Pearson *r* = 0.522, *p* = 0.018), distal diameter (patients: Pearson *r* = 0.536, *p* = 0.015), and volume (Pearson *r* = 0.516, *p* = 0.020) with age.

**Fig. 2:**
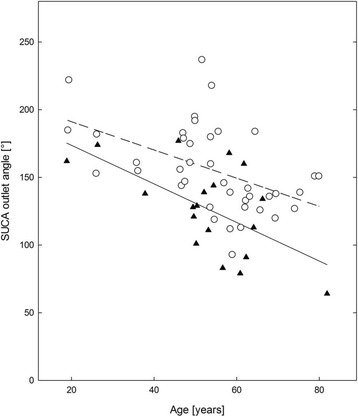
SUCA outlet angle and age in LOPD patients and controls. SUCA outlet angle showed to be age dependent. Assessment of the SUCA outlet angle (in °, sum of left and right) showed to be reduced in higher age in LOPD patients (▲) and matched controls (**○**). Regression lines for LOPD patients (solid line) and for controls (dashed line) are shown

The SUCA outlet angle (Figure 2) was negatively correlating with age in LOPD patients (Pearson *r* = − 0.605, *p* = 0.006) and in controls (Pearson *r* = − 0.483, *p* = 0.002).

In total, in the cohort of all subjects (LOPD patients and controls) the SUCA outlet angle correlated with BA anatomic length (Pearson *r* = − 0,558, *p* < 0.001), the BA linear length (Pearson *r* = − 0.546, *p* < 0.001), the BA volume (Pearson *r* = − 0,545, *p* < 0.001). Correlations according to the Smoker criteria were as follows: with BA diameter (distal: Pearson *r* = − 0.407, *p* = 0.001; middle: Pearson *r* = − 0.363, *p* = 0.005; proximal: Pearson *r* = − 0.280, *p* = 0.032), and the grade of the BA bifurcation height (Pearson *r* = − 0.653, *p* < 0.001) and with the extend of the lateral displacement (Pearson *r* = − 0.287, *p* = 0.028).

There was no gender difference in BA diameter, anatomic length, linear length, volume or SUCA outlet angle in LOPD patients or controls.

### BA characteristics during 5 year follow-up in 9 LOPD patients

At baseline three patients (P8, P10, P16) had an abnormal height of the BA bifurcation. After 5 years two more patients (P3, P6) presented an abnormal BA height. In all 9 patients the individual SUCA outlet angle showed to be decreased after the observation period. On a group level this decrease was significant (Table 3). However, no significant changes were seen for BA diameter, anatomic length, linear length and volume.

**Table 3:** Characteristics of basilar artery in 9 LOPD patients during 5 year follow up measured by TOF-MRA

	Baseline	5 year follow-up^a^	*p* values
Basilar artery characteristics			
- Diameter (mm)			
• proximal • middle • distal	3.8 ± 1.8 3.2 ± 0.7 2.9 ± 0.8	3.6 ± 0.9 3.5 ± 1.5 3.3 ± 1.4	*p* = 0.512 *p* = 0.584 *p* = 0.143
- Anatomic length (mm)	33.2 ± 7.5	33.4 ± 8.2	*p* = 0.808
- Linear length (mm)	30.3 ± 2.9	30.1 ± 3.9	*p* = 0.799
- Volume (ml)	0.214 ± 0.138	0.240 ± 0.211	*p* = 0.475
Smokers criteria			
- Abnormal lateral BA displacement	0/9 (0%)	0/9 (0%)	*p* = 1.00
- Abnormal height of BA bifurcation	3/9 (33%)	5/9 (56%)	*p* = 0.64
Other parameter			
SUCA outlet angle (°)	138 ± 34	128 ± 32	*p* = 0.019
White matter lesions^b^	5/8 (63%)	5/8 (63%)	*p* = 1.00

^a^TOF-MRA was done with the same MR scanner, but different TOF-MR protocols; ^b^one LOPD patient had a CT scan

### Other cerebral abnormalities

During the observation period of 5 years, there were no major cerebrovascular events in 8/9 LOPD patients. However, one of the patients, a 75 year- old female, experienced an intracranial thalamic haemorrhage on the left side that led to complete immobilisation and subsequent death. This patient presented a remarkable dolichoectasia of the BA with an extreme cranial and posteriorly shifting of the BA bifurcation (Additional file 1 Figure S1).

White matter lesions were observed in 12/19 LOPD patients (63%) and 27/40 controls (68%). According to the Fazekas score grading of the LOPD patients was as follows: 40% grade 0, 30% grade 1, 25% grade 2, 5% grade 3. Grading of controls was: 32.5% grade 0, 40% grade 1, 20% grade 2, 7.5% grade 3. On a group level, Fazekas grade was not different in the LOPD patients compared to controls (0.95 ± 0.95 versus 1.05 ± 0.96). In controls, there were no major cerebrovascular abnormalities (e.g. tumor, major stroke, aneurysms).

None of the 9 LOPD patients showed progression of deep white matter lesions in within the 5 year period according to the Fazekas criteria.

The lowest SUCA outlet angle was found in the oldest LOPD patient (P17: 64°), who had a dilated BA and an additional severe mid-basilar stenosis. Other low SUCA outlet angles were found in one patient (P8: 79°) with microbleeds that were exclusively present in the posterior circulation and in one (P14: 83°) with a bilaterally fetal-type circle of Willis.

## Discussion

### Dolichoectasia in patients and controls

The present study on 20 LOPD patients under enzyme replacement therapy (ERT) showed a significant dilation of the BA (regarding diameter and lengths) compared to matched controls. Similar changes have previously reported in 10 of these 20 patients [7]. However, in the present study a more accurate and objective assessment of the vessel diameter was performed using FWHM, based on the pixel-wise intensity [9]. The findings in our cohort are consistent with those of previous studies that found predominant dolichoectasia in the posterior cerebral circulation in LOPD patients with and without ERT [3, 11]. The reason for these changes remains unclear. It was speculated that glycogen deposition in the smooth muscles of the intima of arterial vessels provokes their dilation [2, 12]. In this study all patients received ERT and its impact on cerebral vessels remains enigmatic. However, there is a report that suggests glycogen clearance in smooth muscle via ERT in the arrector pili muscle [13].

However, vertebrobasilar dolichoectasia is not a specific phenomenon in Pompe disease: In general the posterior circulation has less sympathetic innervation than the anterior circulation leading to a predisposition to deformity, when exposed to increased blood flow and pressure [14]. Subsequently, there is physiological progressive luminal dilation, fragmentation of the internal elastic lamina, and elastin loss in aging individuals, that mainly affect the vertebrobasilar arteries [15–17]. Besides a higher age, also a male gender and cardiovascular risk factors (hypertension, smoking, obesity, diabetes, or dyslipidemia) have been found to be associated with evolution of dolichoectasia [18]. Therefore LOPD patients were individually matched with controls of equal epidemiological and cardiovascular parameters.

Since signs of dolichoectasia were observed in both groups, the LOPD patients and the matched controls, additional factors for the development of dilative arteriopathy have to be assumed that result from the comorbidities. However, the BA ectasia was more pronounced in the LOPD cohort compared to controls. It is therefore suggested that Pompe disease acts as independent factor for the development of BA dolichoectasia.

Until now, the most validated parameters for BA evaluation are the semi-quantitative Smoker’s criteria [8]: An abnormal height of BA bifurcation implies that the BA bifurcation is cranial shifted to the level of third ventricle floor or higher. Normally, the SUCA originates shortly under the BA bifurcation at the height of the free edge of the tentorium cerebelli [19] and the SUCA outlet angle is nearly 180°. If the SUCA origin and the adjacent basilar bifurcation shift cranial, then the SUCA outlet angle will consequently decrease. The SUCA is most consistent and the angle is easily obtained from coronal CT- or MR angiography images, independently from different imaging protocols. Therefor in this study the outlet angle of the SUCA was introduced as a novel parameter for a quantitative analysis of this cranial shift of BA bifurcation. It was demonstrated that the SUCA outlet angle in LOPD patients was significantly reduced compared to controls together with other indicators of a prominent BA ectasia in these patients. Additionally, there was a significant correlation of the SUCA outlet angle with all parameters of the Smoker’s criteria: BA diameter, bifurcation height and lateral displacement.

### Analysis of progression of dolichoectasia in LOPD patients

In the present study, according to the Smoker’s criteria [8], only 3/9 LOPD patients showed an abnormal height of the BA bifurcation at baseline but 5/9 LOPD patients after 5 years follow up. In the 5 year follow up analysis there was a significant decline of the SUCA outlet angle in the cohort of 9 LOPD patients on ERT that suggests a progressive dolichoectasia in these patients. The higher slope of the regressionline of the LOPD cohort compared to controls in the age dependent analysis of the SUCA outlet suggests that these differences result from disease specific mechanism rather from the aging of the patients.

However, other parameters that define BA dolichoectasia (length, diameter, and lateral displacement of the BA) remained nearly unchanged. Similar results were reported in a recent study showing stable vertebrobasilar diameters over a period of up to 10 years in 5 LOPD patients [20]. This might be due to the relatively small diameter and lengths of the BA. Changes of these parameters may need time to evolve and are therefore not easily detectable in the 5 year observation period. However, presumed that all vertebrobasilar arteries are affected by a weakening of the vessel wall, and then the cranial migration of the BA bifurcation might be a result of a summation effect of the elongation of the BA plus the adjacent vertebral arteries. Since vasodilation results in reduced flow velocity, the dilation of cerebral arteries might be underestimated in TOF-based MRA. Limitation of this study might be the use of slight differently TOF-MRI protocols in the follow up cerebral imaging. However, this has no impact on the SUCA outlet angle. It is suggested that the SUCA outlet angle is a sensitive surrogate marker for BA dolichoectasia, which detects the elevation of the height of basilar bifurcation in a more precise and quantitative way than the Smoker’s grading.

### Clinical relevance

It is well known that dolichoectasia can lead to brainstem compression, hydrocephalus microbleeds and stroke [18, 21, 22]. There are several reports on strokes [6, 23] and intracranial mirco – and macrohemorrhages [4, 5, 24] in Pompe patients. In the actual LOPD cohort a fatal thalamic hemorrhage occurred in one patient with the most prominent basilar dolichoectasia. It can be speculated, that the vertebrobasilar pathology might at least has contributed to the hemorrhage in this patient. However, in all 9 patients there was no progression of white matter lesions after the follow up of 5 years.

It seems therefore appropriate, that patients without cerebrovascular abnormalities undergo a follow up of MRI analysis after 5 years. In patients with cerebrovascular abnormalities, we recommend the strict treatment of cerebrovascular risk factors (e.g. hypertension) and follow up imaging after 1 to 2 years.

## Conclusion

Dilation and elongation of the BA is pronounced in LOPD patients under ERT compared to matched controls. This suggests that LOPD acts as an independent risk factor for basilar dolichoectasia. The study demonstrates a decreasing SUCA outlet angle as surrogate marker for a progressive elevation of the BA bifurcation in the disease course of LOPD during a 5 year observation period. The SUCA outlet angle seems to be useful for detection of progression of vertebrobasilar dolichoectasia.

## Additional file

Additional file 1: **Figure S1.** A case of very prominent vertebrobasilar dolichoectasia. Vertebrobasilar arteries of a 75 year old female LOPD patient. Basilar artery shows to be dilated and elongated with massive cranial and posterior shift of the BA bifurcation height (A coronal, B sagittal view). This LOPD patient experienced a left side thalamic hemorrhage. As consequence of massive BA shift the SUCA outlet angle was not reasonable measurable. Abbr. 1 distal part of basilar artery, 2 bifurcation of basilar artery, 3 posterior cerebral artery, 4 superior cerebellar artery, 5 anterior inferior cerebellar artery, 6 vertebral artery. (PDF 150 kb)

## Abbreviations

BA: Basilar artery
ERT: Enzyme replacement therapy
LOPD: Late onset Pompe disease
SUCA: Superior cerebellar artery
VA: Vertebral artery
