# Supplementary figures and images for: Decreased outlet angle of the superior cerebellar artery as indicator for dolichoectasia in late onset Pompe disease
Source: Orphanet J Rare Dis. 2018 Apr 13;13:57. doi: 10.1186/s13023-018-0794-6 (PMC5899367; doi:10.1186/s13023-018-0794-6)

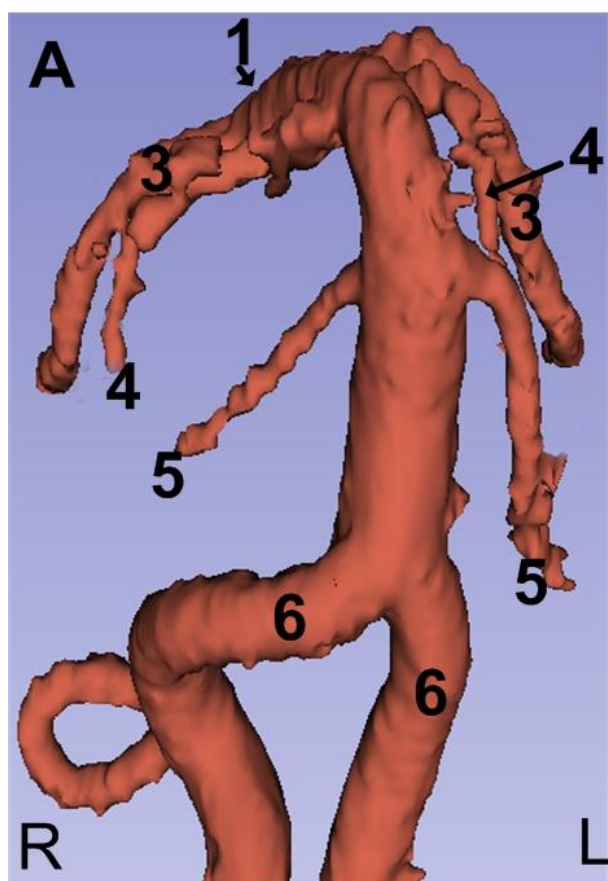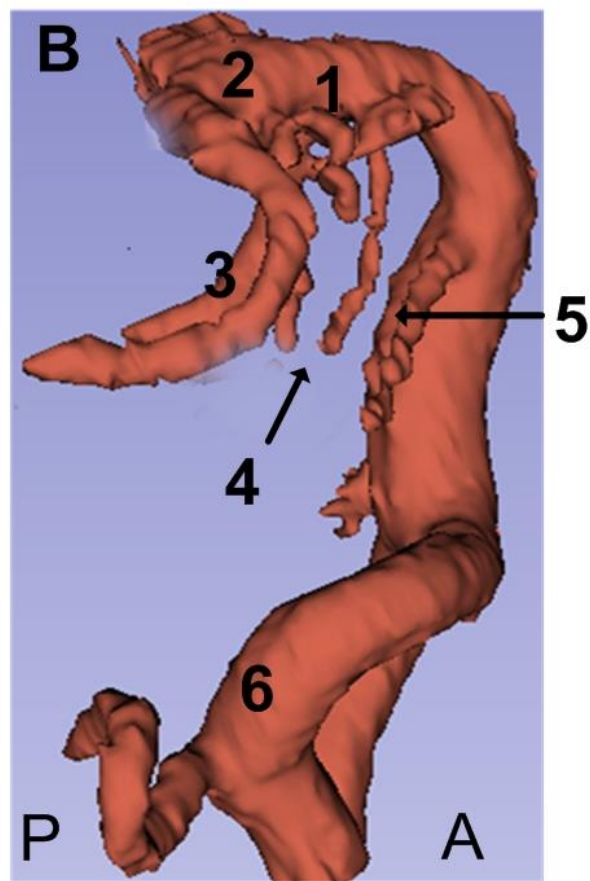

Supplement: Supplementary file 1 — Figure S1. A case of very prominent vertebrobasilar dolichoectasia. Vertebrobasilar arteries of a 75 year old female LOPD patient. Basilar artery shows to be dilated and elongated with massive cranial and posterior shift of the BA bifurcation height (A coronal, B sagittal view). This LOPD patient experienced a left side thalamic hemorrhage. As consequence of massive BA shift the SUCA outlet angle was not reasonable measurable. Abbr. 1 distal part of basilar artery, 2 bifurcation of basilar artery, 3 posterior cerebral artery, 4 superior cerebellar artery, 5 anterior inferior cerebellar artery, 6 vertebral artery. (PDF 150 kb) [file 13023_2018_794_MOESM1_ESM.pdf]
